# An Easy-Made, Economical and Efficient Carbon-Doped Amorphous TiO_2_ Photocatalyst Obtained by Microwave Assisted Synthesis for the Degradation of Rhodamine B

**DOI:** 10.3390/ma10121447

**Published:** 2017-12-20

**Authors:** Adan Luna-Flores, José L. Sosa-Sánchez, Marco Antonio Morales-Sánchez, Ricardo Agustín-Serrano, J. A. Luna-López

**Affiliations:** 1 Facultad de Ingeniería Química-BUAP, Avenida San Claudio, Ciudad Universitaria, Puebla 72570, Mexico; adan.luna@correo.buap.mx (A.L.-F.); spinor70@yahoo.com.mx (M.A.M.-S.); 2 Centro de Investigación en Dispositivos Semiconductores, Instituto de Ciencias BUAP, 14 sur y Avenida San Claudio, Ciudad Universitaria, A.P. 196, Puebla 72000, Mexico; jose.luna@correo.buap.mx; 3 Centro Universitario de Vinculación y Transferencia de Tecnología OTC-BUAP, Prolongación de la 24 Sur y Avenida San Claudio, Ciudad Universitaria, Puebla 72570, Mexico; ricardoagustin_s@hotmail.com

**Keywords:** amorphous titanium oxide, microwave assisted synthesis, Triple-E photocatalyst

## Abstract

The search for novel materials and the development of improved processes for water purification have attracted the interest of researchers worldwide and the use of titanium dioxide in photocatalytic processes for the degradation of organic pollutants contained in water has been one of the benchmarks. Compared to crystalline titanium dioxide (cTiO_2_), the amorphous material has the advantages of having a higher adsorption capacity and being easier to dope with metal and non-metal elements. In this work, we take advantage of these two features to improve its photocatalytic properties in the degradation of Rhodamine B. The structural characterization by XRD analysis gives evidence of its amorphous nature and the SEM micrographs portray the disc morphology of 300 nm in diameter with heterogeneous grain boundaries. The degradation of Rhodamine B tests with the amorphous TiO_2_ using visible light confirm its improved catalytic activity compared to that of a commercial product, Degussa P25, which is a well-known crystalline material.

## 1. Introduction

The possibility of putting on the market TiO_2_-based products is a potentially profitable area of nanostructured materials development due to the advantageous properties of the material (low price, chemical inertness, long-term stability, lack of toxicity, etc.) and its different practical uses [1]. TiO_2_ industrial use can now be seen in various applications that include self-cleaning surfaces, anti-fogging mirrors, antimicrobial coatings, electrochromic devices, rechargeable batteries, and sensors. More practical applications in medical, automotive, and photocatalytic environmental remediation of water and air can also be forecasted for the near future [2,3,4].

For these applications, crystalline TiO_2_ (cTiO_2_) in its different phases (anatase, rutile, and brookite) has been extensively studied even with some DFT theoretical calculations [5], in particular for its electronic structures and defect levels. On the other hand, the studies on the amorphous TiO_2_ (aTiO_2_) are not enough [6], even though it is the amorphous state that sometimes plays crucial roles in particular applications.

The choice of crystalline TiO_2_ in the anatase phase as the most active material is justified only for applications where its activity does not depend on the optical and electronic properties of the TiO_2_ (self-cleaning and anti-fogging). However, for other uses that include photovoltaic devices and photocatalytic processes the crystalline material has a great disadvantage due to its wide bandgap (3.2 eV), since this makes necessary the use of ultraviolet radiation for its activation, and the sun has only 4–5% of this radiation component. Other drawbacks of cTiO_2_ are related to the synthetic simplicity and reproducibility to obtain the desired product, and here, the amorphous material can be a good alternative.

Among the well-established traits of aTiO_2_, its facile large-scale production, higher surface area, and its concomitant higher absorptivity [7] are worth mentioning. In addition, aTiO_2_ can form soft interfaces with substrates with different lattice parameters, and due to its isotropic nature, its optical properties do not depend on its orientation.

Owing to these favorable properties, aTiO_2_ has been used to stabilize photoanodes for an efficient water splitting process [8] and to improve the performance of Dye Sensitized Solar Cells [9]. In addition, other recent reports [10] have shown that aTiO_2_ may match the photocatalytic activity of cTiO_2_. Finally, some other theoretical (DFT + U) studies [11,12] suggest that aTiO_2_ may be more suited than cTiO_2_ for doping or impurification to broaden its absorptivity into the visible range. Several approaches have been developed to accomplish this: coupling of other narrow band gap semiconductors (CdS, CdSe), surface sensitization with dyes and metals (Cr, Fe, Mn, V), and nonmetal doping (N, C, F, B) [13].

In this context, carbon doping has aroused some research interest since this element can be incorporated to the lattice in different ways: as an anion (substituting the oxygen atom [14]), as a cation occupying interstitial sites, or even substituting titanium atoms [15]. It can also be integrated in a composite TiO_2_-based catalyst as a thin layer [16,17,18] or decorating the surface of the oxide material [19]. Regarding the synthetic feasibility, carbon doping can be carried out “in situ” in a one-pot reaction without using an external carbon precursor [20].

In this report, we aimed to prepare a Triple-E Carbon Doped amorphous titanium dioxide (CD-aTiO_2_) that is easy-to-make using microwave dielectric heating, economical because it does not require an external carbon precursor, and more efficient than the commercial cTiO_2_ Degussa P25 for the photocatalytic degradation of Rhodamine B.

The sequence of simple steps for the greener microwave-assisted one-pot synthesis of CD-aTiO_2_ using commercially available reagents and the fastness of the procedure accounts for the easy-to-make and economical labels. In addition, no external source of carbon was used for the same synthetic procedure. A higher photocatalytic activity for the degradation of Rhodamine B compared to that of commercial Degussa P25 cTiO_2_ is demonstrated for the carbon doped aTiO_2_ and this justifies the efficient catalyst label.

## 2. Results and Discussion

### 2.1. Morphology, Short-Range Crystalline Structure and Surface Characterization

**Figure 1:**
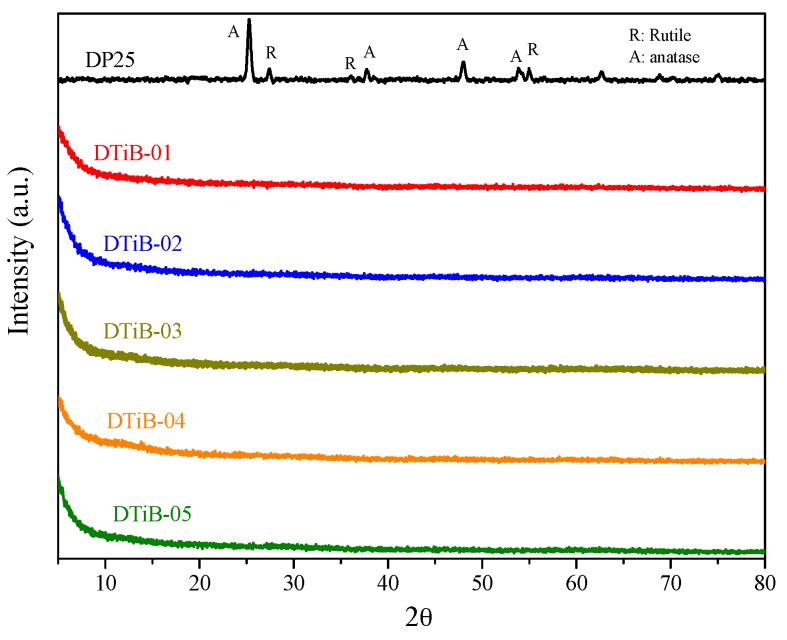
X-ray diffractograms of all the obtained aTiO_2_ and commercial DP25 products.

The XRD diffraction patterns for all the CD-aTiO_2_ materials obtained in this work are shown in Figure 1a, and the most apparent feature is their amorphous nature, portrayed by the flat appearance of all the diffractograms [21,22]. No sharp diffraction peaks were obtained for any of the products. As expected, the diffraction pattern for the commercial DP25 TiO_2_ shown at the top in Figure 1 exhibits the diffraction peaks for the anatase and rutile phases present in this material.

**Figure 2:**
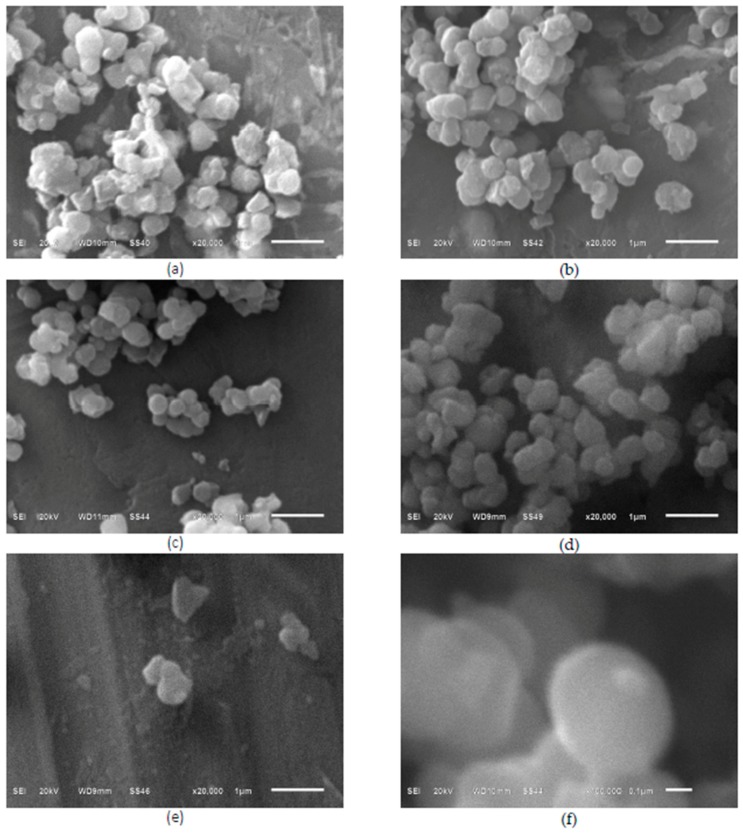
Scanning electron microscope (SEM) micrographs of all the CD-aTiO_2_ prepared (**a**) DTIB-01; (**b**) DTIB-02; (**c**) DTIB-03; (**d**) DTIB-04; (**e**) DTIB-05; (**f**) the disc morphology with a dimension of ~300 nm in diameter of these products can be observed in the last pictured.

In Figure 2, a uniform grain size distribution can be seen for all the SEM images of the solid TiO_2_ products. The disc-shaped particles of nanometric dimensions with a ~300 nm radius form conglomerates of bigger dimensions. Kojima and Sugimoto [23] have shed some light into the particle formation mechanism under similar synthetic protocols where the effects of water content, temperature, and solvent composition were studied.

Although the formation of carbon-doped TiO_2_ nuclei to obtain nanocrystals has been described previously [24], for our solution method assisted with microwave radiation no reports were found and therefore, in the following text we suggest a plausible mechanism of nano particle formation taking into account its morphology. In order to shed some insight on the particle formation mechanism, we have to consider that in the microwave-assisted synthesis, an in situ generation of heat gives rise to inverted temperature gradients compared with the conventional heating methods. This might generate a concentration gradient which in turn may break the crystal symmetry during its growing. In addition, the incorporation of carbon during the microwave heating process may result in a decrease of the particle size. Taking into account these two aspects, we propose that the preferential growing of the particle would be in the radial direction to form nanodiscs of CD-aTiO_2_. The heterogeneous grain boundaries observed for the particles from the SEM micrographs is determined by the pH of the reaction mixture [25].

The disc morphology of the TiO_2_ photocatalysts can reduce the migration distance of charge carriers when they travel through its thickness (~20 nm) and therefore may be considered to account for the increased transport of charge carriers in the amorphous photocatalysts obtained in this work and their concomitant higher photocatalytic activity.

**Figure 3:**
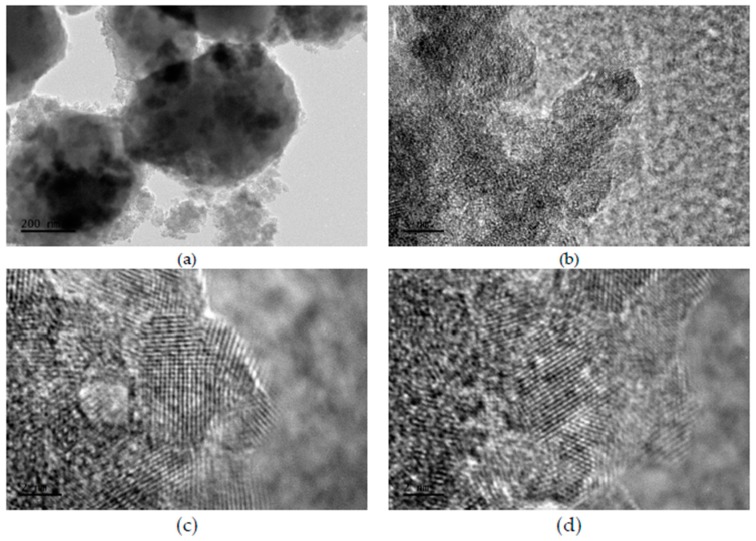
Transmission electron microscope (TEM) images of carbon-coated CD-aTiO_2_ for the DTiB-04 photocatalyst, (**a**) particles conglomerates at 200 nm (see Figure 1b), (**b**) zoom of image (**a**) to 5 nm and (**c**,**d**) nanocrystals of image (**b**) to 2 nm.

In Figure 3a, the micrographs obtained from a transmission electron microscope show the particles conglomerates of CD-aTiO_2_ at 200 nm. The particles having dark contrast in the photo are supposed to be TiO_2_ particles. These particles are surrounded by some thin layers with faint contrast, which are believed to be the carbon dopant.

**Figure 4:**
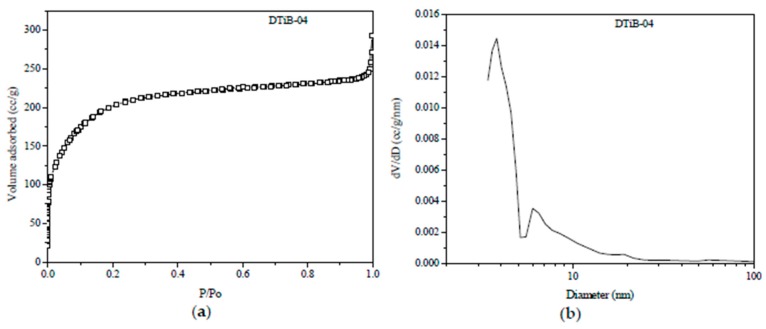
(**a**) N_2_ adsorption-desorption plot for the DTiB-04 isotherm; (**b**) Pore size distribution curve for the same DTiB-04 sample obtained from the desorption isotherm.

Figure 3b is a zoom of image 3a, and in this micrograph, the agglomeration and connection of adjacent nanoparticles through carbon layers are exhibited, but only for the sample DTiB-04 as an example. Finally, Figure 3c,d is zooms of two different regions of image 3b in a 2 nm scale. These photos show the surfaces of TiO_2_ nanocrystals, where the disordered outer layer surrounding a crystalline core can be clearly observed with a faint contrast. Particles conglomerates of carbon-coated CD-aTiO_2_ have been obtained using other synthetic methods [18,22,26]. In these work, the role of carbon is used to explain the amorphous structure of CD-aTiO_2_. The carbon is incorporated as a layer covering the nanocrystals, which can be doped or not doped with this element. Figure 2c,d shows evidence of coated and doped carbon nanocrystals. The adsorption-desorption isotherm curve for the DTiB-04 material is shown in Figure 4a.

In the Figure 4a, we can observe that the isotherm for the DTiB-04 sample exhibits a characteristic Langmuir behavior (type II according to the BDDT classification) and its features have been associated with functional materials that include activated carbon and porous oxides [27].

In Figure 4b where the pore-size distribution of the same sample is shown we can observe two different kinds of pores for the same amorphous material. The one corresponding to the region centered at 3.8 nm is associated to the mesopores on the amorphous catalyst surface, and the other centered at 6 nm should correspond to those cavities originated from the conglomeration of CD-aTiO_2_ particles [18]. A worth noting feature obtained from the N_2_ isotherms data is the relatively high surface area S_BET_ = 760 m^2^/g compared to that of other amorphous TiO_2_ catalysts < 500 m^2^/g [7,10,18,22]. This 50% increase of the surface area in our DTiB-04 catalyst is created by the carbon surface layer (the TEM micrographs confirm this) incorporated during the microwave heating treatment and accounts for the high adsorption capacity of the same material in the dark stage. 

### 2.2. FT-IR Analysis

**Figure 5:**
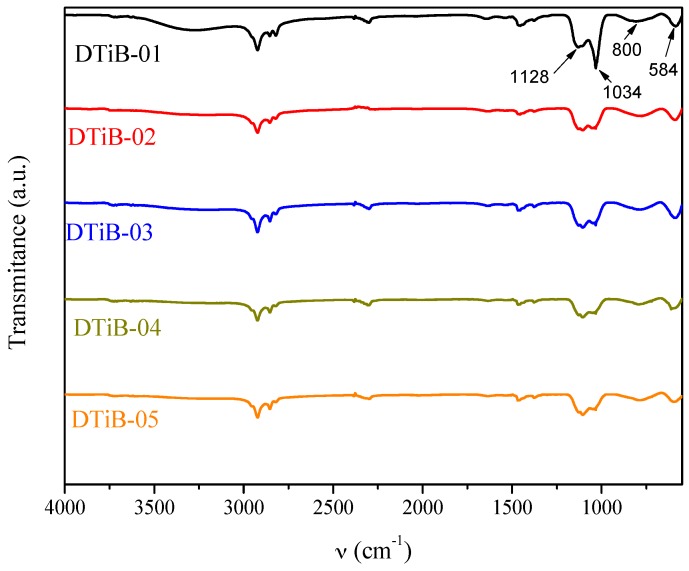
Fourier-transform infrared spectroscopy (FT-IR) spectra for all the CD-aTiO_2_ synthetized and used in this work.

The FT-IR spectra of all the CD-aTiO_2_ obtained are shown in Figure 5. The vibrational modes at 1128 and 1034 cm^−1^ are assigned to the Ti-OH bond. In addition, the peak centered at 584 cm^−1^ that appears in the spectra corresponds to the O-Ti-O stretching vibration [28]. The broad absorption at 800 cm^−1^ has been associated to the incorporation of carbon [22,29].

### 2.3. Thermogravimetric (TG) and Differential Scanning Calorimetry (DSC) Analyses

**Figure 6:**
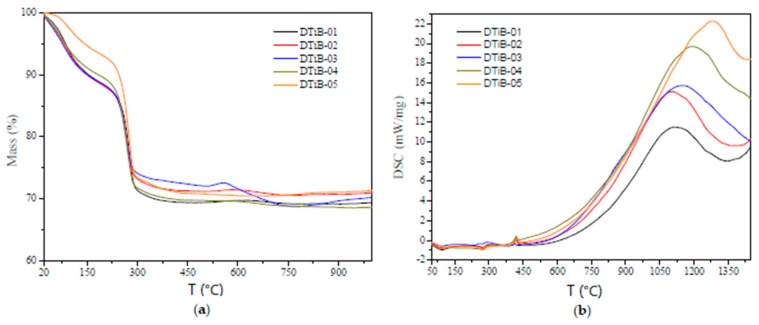
(**a**) Thermogravimetric (TG) and (**b**) differential scanning calorimetry (DSC) analysis for all the CD-aTiO_2_ photocatalysts synthetized and used in this work.

The TG and DSC plots are shown in Figure 6. The first 10% weight loss observed in Figure 6a, from the very beginning, between room temperature and 110 °C in the TG curve is attributed to the evaporation of water and the solvents used for rinsing the photocatalysts. The presence of a small broad signal around 90–100 °C in the DSC plots confirms the occurrence of this physical process. Then, a second weight loss detected in the TG curves in the temperature range of 230–290 °C corresponds, in our view, to the loss of organic matter (mainly 1-decanol) absorbed in the TiO_2_ photocatalysts. Here again, the other broad small signal centered around 270 °C in the DSC analysis confirms such loss. Above 300 °C no weight loss can be seen in the TG analysis. However, in the DSC curves additional signals attributed to two phase changes of the amorphous material are observed. The first phase change occurs at 420 °C when the amorphous material is transformed to the anatase polymorph. For the second phase transformation, from anatase to rutile, occurring above 600 °C for all the synthetized TiO_2_ materials, a shift to the right in the maxima of the endothermic peaks (corresponding to the phase transformations) can be observed in Figure 6b. This can be accounted for by considering, according to Palanivelu et al. [30] and Enache et al. [31], the different carbon contents acquired during the microwave heating step of all the carbon-doped materials. In addition, since no weight loss is observed in the TG analysis after the 300 °C threshold, we assume that the carbon dopant is incorporated to the TiO_2_ lattice as can be observed in the TEM images. This incorporation of carbon was confirmed by EDS and TEM analysis, and the compositional results are shown in Table 1.

The EDS compositional analysis (see Figure S1 of the supplementary information) for the CD-aTiO_2_ samples (included in the last three columns of Table 1) show that the amount of carbon incorporated into the nanocrystals is determined by the irradiation time during the microwave treatment. Under these experimental conditions, after six minutes of irradiation, the DTiB-04 catalyst incorporated the biggest amount of carbon compared to the other catalysts and at the same time favored the short range order and crystallinity (see Figure 3 and Figure 6b).

**Figure 7:**
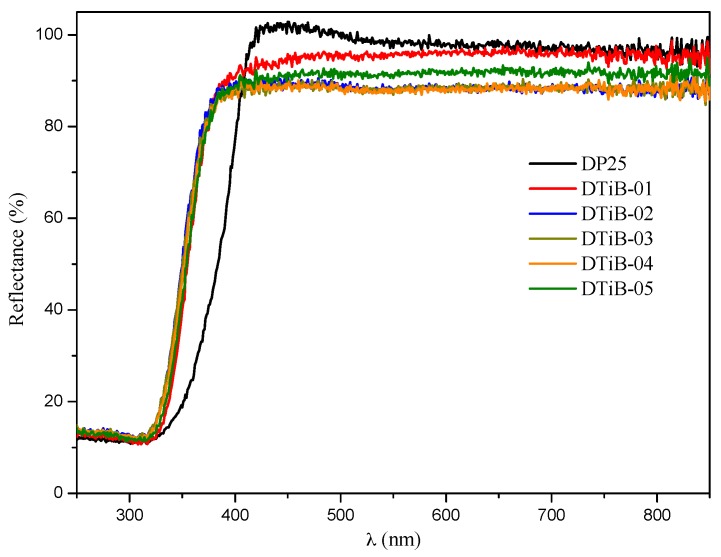
Diffuse reflectance ultraviolet–visible spectroscopy (DRS UV-vis) spectra for all the CD-aTiO_2_ samples prepared in this work and for the commercial DP25 TiO_2_.

The diffuse reflectance UV-vis spectra for all the prepared samples are shown in Figure 7. No significant difference can be observed for all the curves corresponding to the CD-aTiO_2_ photocatalysts compared to the DP25 commercial product, and consequently, the band gap values obtained from them using the Kubelka-Munk theory [32] are very similar, ranging from 3.37–3.41 eV for the amorphous materials (see Figure S2 of the supplementary information).

### 2.4. Photocatalytic Degradation of Rhodamine B Using the TiO_2_ Products

In the procedure followed for the degradation of Rhodamine B, the initial step is the adsorption of Rhodamine B onto the TiO_2_ photocatalyst surface, and this was monitored using UV-vis spectrophotometry (see Figure S3 of supplementary information). Therefore, with the aim of reaching the adsorption–desorption equilibrium, the photocatalyst is first dispersed into the dye solution using an ultrasonic bath and later, a current of air is bubbled through the liquid to disperse homogeneously the solid photocatalyst in the dark for a 20 min period. The spectra for the adsorption process to reach this equilibrium for all the photocatalysts used in this work are portrayed in Figure 6, and in Table 1, the adsorption percentages for each test with respect to the initial concentration of colorant are given.

**Figure 8:**
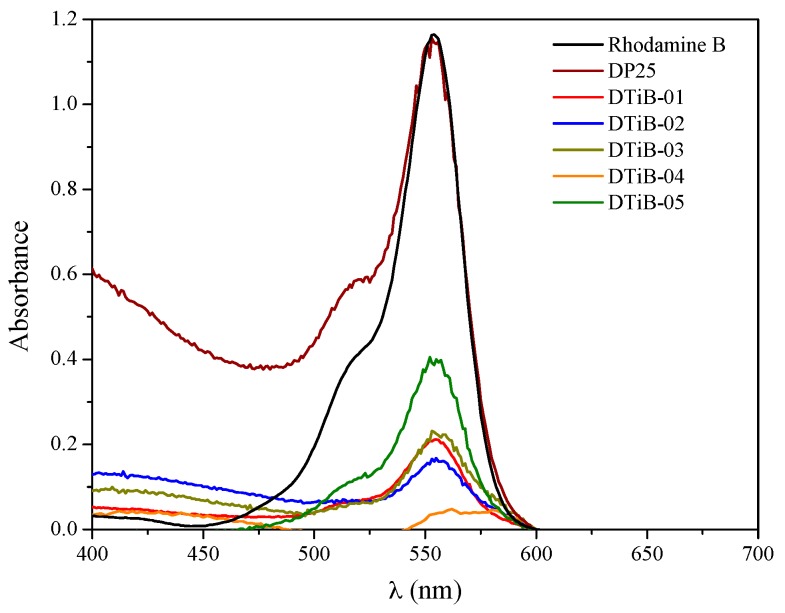
UV-Vis spectra of the final concentration of Rhodamine B after a 20 min adsorption process in the presence of all the photocatalysts.

A significant decrease of the concentration of the Rhodamine B solution due only to the adsorption process can be observed in Figure 8, and this has been reported by Kanna et al. [7]. 

Another interesting feature detected from the UV-vis spectra during the adsorption process is a significant shift of the absorption peak only for the DTiB-04 photocatalyst and a concomitant broadening of the absorption bands [33] for all the Rhodamine B solutions monitored during the 20 min adsorption process, which can also be observed in Figure 8. In Table 1, the wavelength of these maxima in the UV-vis spectra after 20 min of adsorption are listed.

As mentioned above, the disc morphology of the TiO_2_ photocatalysts can favor the transport of charge carriers from the bulk to the surface of the amorphous photocatalysts by reducing their migration distance when they travel through its thickness (~20 nm), resulting in a higher photocatalytic activity.

In relation to the degradation mechanism, it is well known that the sequential loss of the four N-ethyl groups occurs in the first stages.

**Figure 9:**
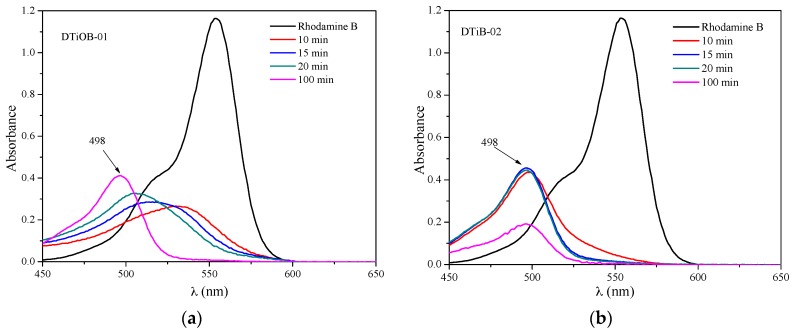
Hypsochromic shift detected during the degradation process of Rhodamine B with (**a**) DTiB-01 and (**b**) DTiB-02.

**Figure 10:**
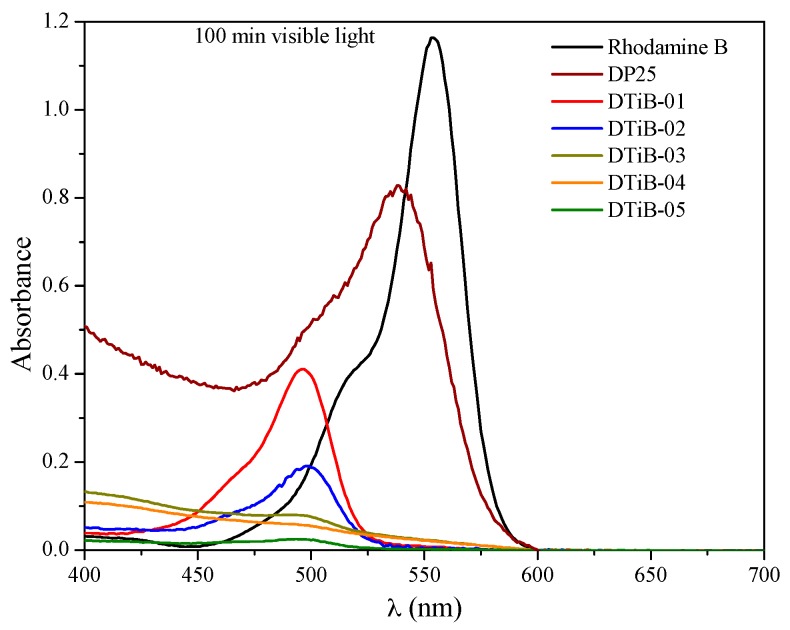
UV-Vis spectra for the remaining Rhodamine B solutions after a 100 min exposure to visible light.

When the Rhodamine B dye is irradiated in the presence of TiO_2_ photocatalysts, the consecutive loss of the N-ethyl groups occurs, as has been reported previously. In our case, for the DTiB-01 and the DTiB-02 photocatalysts, the UV-Vis spectra show this loss by exhibiting a hypsochromic effect of the absorptions to the 498 nm wavelength (see Figure 9) in accordance to the report of Rochkind et al. [33]. For these two catalysts, the complete degradation of the dye does not happen, since their absorption peaks corresponding to the base-structure are still present after the degradation process. For the remaining photocatalyst samples, DTiB-03, 04 and 05 the corresponding peaks at 498 nm almost disappear after 100 min of visible light irradiation, as can be observed in Figure 10, and this suggests that the degradation process was accomplished effectively.

**Figure 11:**
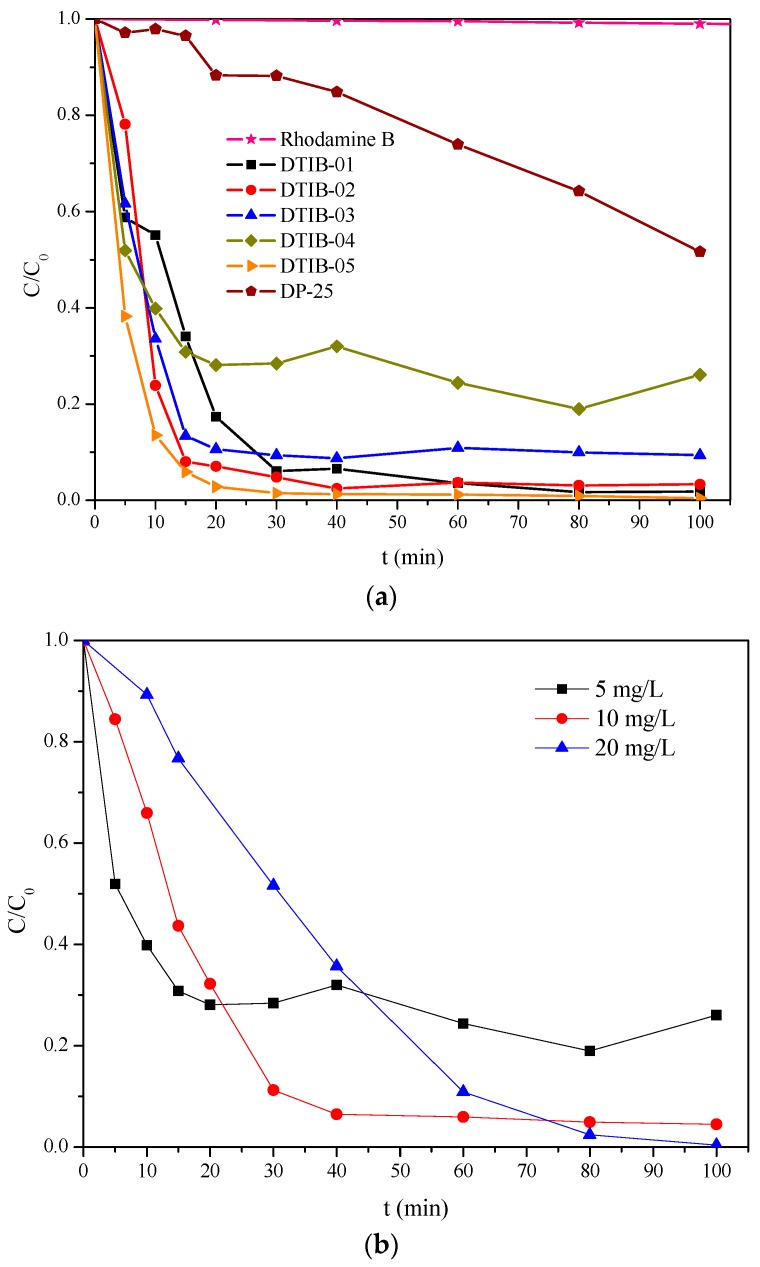
(**a**) Adsorption (in the dark) and degradation (during exposure to visible light) rates of Rhodamine B for all the obtained photocatalysts; (**b**) Adsorption and degradation rates for increasing concentrations of Rhodamine B using the same DTiB-04 photocatalyst.

The Rhodamine B photolysis and the degradation rate plots are presented in Figure 11a. Here, the dye concentration remains constant even after a 100 min irradiation period with visible light indicating that under this condition photolysis does not occur as reported previously by Wu et al. [34]. To make a better comparison of the photocatalytic activity for all the samples a normalization of the Rhodamine B concentration was considered taking its concentration, after its absorption in the dark, as reference. In Figure S4 of the supplementary information the the kinetic data for the Rhodamine B degradation process are shown. Another conspicuous feature observed from the same Figure 11a is the different absorption and degradation rates for the DTiB-04 catalyst compared to those of the others after a 60 min exposure with visible light. For the DTiB-01, 02 and 05 samples, a 96% was accomplished in contrast to the 76% value obtained for the DTiB-04 photocatalyst, which also exhibits the highest absorption during the dark stage. In our view, it is apparent that this increased absorption of the dye onto the DTiB-04 catalyst surface blocks the active sites available for the degradation process resulting in a decreased degradation rate for the whole process. To verify this assumption, we carried out other tests in which the original concentration of the dye was varied (vide infra). In addition, the efficiency of all the CD-aTiO_2_ products obtained here for the degradation of Rhodamine B is quite high (more than 90%) compared to that of the commercial DP25 titanium oxide (no more than 50%) after the 100 min period. The photocatalytic activity of the latter under visible light irradiation has been accounted for in previous reports [34,35], and it is attributed to the sensitization activity of the Rhodamine B by itself [36].

Since the adsorption capacity for all the CD-aTiO_2_ catalysts was quite high, a reasonable doubt was cast with respect to the degradation activity, in particular if higher concentrations of Rhodamine B dye were used. Here, we assumed that increasing the concentration of the dye the reaction kinetics for the decomposition process on the catalysts surface may be affected by the blocking of their active sites in the presence of high concentrations of Rhodamine B, as we already suggested in a previous paragraph. To confirm this, the degradation activity of the CD-aTiO_2_ photocatalysts after the adsorption of higher amounts of Rhodamine B onto their surface two more concentrated solutions were tested (10 and 20 mg/L) only for the DTiB-04 catalyst which contains a higher percentage of carbon and therefore exhibits a higher adsorption percentage. In Figure 11b, it is apparent that, although only a small amount of Rhodamine B is eliminated from the more concentrated solutions (10 and 20 mg/L) after the adsorption in the dark, later in the presence of light, the photocatalyst can degrade the dye. In other words, the initial adsorption process does not interfere with the subsequent photocatalytic degradation. According to our results, when the DTiB-04 catalyst is used during the degradation process of the more concentrated solutions, all the remaining dye is degraded in a relatively short time. This confirms the increased photocatalytic activity of the CD-aTiO_2_ materials compared to the commercial DP25 product despite their amorphous structure. 

We rationalize that the nanodisc morphology of the CD-aTiO_2_ materials favors the catalytic degradation mechanism in a similar fashion to that reported by Shao et al. [22], as mentioned above.

## 3. Materials and Methods

### 3.1. Materials and Reagents

All commercially available reagents were used without further purification and their brand names are as follows: 1-decanol (99.8%, EMD Millipore, Burlington, MA, USA), titanium butoxide (97.0%, Aldrich, Saint Louis, MO, USA), acetone (99.5%, J.T. Baker, Phillipsburg, NJ, USA), and methanol (99.8%, J.T. Baker, Phillipsburg, NJ, USA). Deionized water obtained from a Millipore system was used to prepare all the solutions for the photocatalytic experiments.

### 3.2. Synthesis of Carbon-Doped Amorphous Titanium Dioxide (CD-aTiO_2_)

The synthesis of CD-aTiO_2_ was carried out as follows: in a round bottom 100 mL flask 15 mL of 1-decanol were heated using a hotplate with magnetic stirring for 10 min at 80 °C and then 2 mL of titanium butoxide were added under vigorous stirring maintained for other 10 min. Then, 4 mL of deionized water were added, and the mixture was stirred 7 min more. At this stage, microwave heating was provided during 0, 2, 4, 6, and 8 min to yield a series of photocatalysts labelled DTiOB-01, DTiOB-02, DTiOB-03, DTiOB-04, and DTiOB-05, respectively. The greyish-white solid products obtained were rinsed with hot acetone first and then with hot methanol to get rid of any by-products, and then they were dried at 80 °C for 30 min.

### 3.3. Characterization and Analytical Techniques

XRD analysis was performed using a Bruker X-ray Diffraction D6-Discover equipment (Bruker, Billerica, MA, USA), Diffuse Reflectance Spectroscopy (DRS) using a Varian Cary 400 spectrophotometer (Cary, Addison, IL, USA) with a Harrick RD accessory (Harrick, Ithaca, NY, USA) was used for the optical characterization of the samples. The FT-IR spectra were obtained using a Perkin Elmer Spectrum (Perkin Elmer, Waltham, MA, USA), one spectrophotometer equipped with an Attenuated Total Reflection (ATR) accessory. The SEM micrographs were obtained with a JSM-6610LV JEOL electron microscope (JEOL, Akishima, Japan), and the transmission electron microscopy (TEM) with a Philips Tecnai F20, 200 kV (Philips, Amsterdam, The Netherlands). The nitrogen adsorption analysis was carried out to determine pore size distribution and the isotherm curve using an Autosorb 1C Quantachrome (Quantachrome, Boynton Beach, FL, USA) equipped with a Verlab VE-5600UV photometer (Quantachrome, Boynton Beach, FL, USA) and MetaSpec Pro analysis software (Quantachrome, Boynton Beach, FL, USA).

### 3.4. Measurements of Photocatalytic Activity

The photocatalytic activity tests were carried out using a previously described system [36] that uses a 10 W LED visible light source with a spectral power distribution in the 500–800 nm range (see Figure S5 of the supplementary information). For each degradation test, 15 mg of the TiO_2_ photocatalyst were dispersed in 60 mL of a Rhodamine B solution prepared at different concentrations (5, 10, and 20 mg/L) for one minute using an ultrasonic bath. The suspension was maintained in the dark under stirring conditions for 20 min to reach the adsorption-desorption equilibrium, and the degradation processes were monitored by UV-vis spectroscopic analysis from samples taken at regular time intervals to complete 100 min. 

The influence of the carbon content and crystallinity of the samples on the photocatalytic activity was verified by carrying out additional tests: a) the comparison of the photocatalytic activity of the carbon-doped DTiB-04 catalyst taking as a reference a non-doped material (DTiB-06), and b) the comparison of the photocatalytic performance of the amorphous DTiB-05 material with its crystalline derivative obtained by thermal treatment above 500 °C (DTiB-05TT). Figures S6 and S7 of the supplementary information show the results obtained.

## 4. Conclusions

An improved methodology (easy-to-make and economical) for the synthesis of amorphous carbon-doped TiO_2_ assisted by microwave heating and without using an external carbon source was developed. The nanodisc amorphous morphology, determined by XRD, SEM, and TEM analysis for all the photocatalysts obtained here, enhances their photocatalytic activity. Compared to that of commercial crystalline TiO_2_, it is significantly higher (more efficient). This high photocatalytic activity (close to 100%) for all the CD-aTiO_2_ products was kept even at higher concentrations of Rhodamine B (four times higher) after the 100 min degradation process.

## Figures and Tables

**Table 1:** Some parameters of the characterization results for all the CD-aTiO_2_ photocatalysts obtained under different microwave heating conditions.

TiO_2_ Photocatalyst	Microwave Heating (t, min)	Eg (eV)	Adsorption (%)	λ_max_ (nm) in the UV-Vis Analysis	C (%w)	O (%w)	Ti (%w)
DP25	0	3.10	1.3	554	-	-	-
DTiB-01	0	3.37	80	555	21.22	38.34	40.43
DTiB-02	2	3.41	84	555	20.75	40.04	39.21
DTiB-03	4	3.40	78	556	23.56	39.99	36.55
DTiB-04	6	3.39	95	563	24.29	40.11	35.60
DTiB-05	8	3.38	60	554	22.04	37.10	40.87

## Supplementary Materials

The following are available online at www.mdpi.com/1996-1944/10/12/1447/s1.

## References

[1] Ramos-Delgado N.A., Gracia-Pinilla M.A., Mangalaraja R.V., O’Shea K., Dionysiou D.D. (2016). Industrial synthesis and characterization of nanophotocatalysts materials: Titania. Nanotechnol. Rev..

[2] Low J., Cheng B., Yu J. (2017). Surface modification and enhanced photocatalytic CO_2_ reduction performance of TiO_2_: A review. Appl. Surf. Sci..

[3] Bai S., Jiang J., Zhang Q., Xiong Y. (2015). Steering charge kinetics in photocatalysis: Intersection of materials syntheses, characterization techniques and theoretical simulations. Chem. Soc. Rev..

[4] Ma Y., Wang X., Jia Y., Chen X., Han H., Li C. (2014). Titanium Dioxide-Based Nanomaterials for Photocatalytic Fuel Generations. Chem. Rev..

[5] Landmann M., Rauls E., Schmidt W.G. (2012). The electronic structure and optical response of rutile, anatase and brookite TiO_2_. J. Phys. Condens. Matter..

[6] Landmann M., Kohler T., Koppen S., Rauls E., Frauenheim T., Schmidt W.G. (2012). Fingerprints of order and disorder in the electronic and optical properties of crystalline and amorphous TiO_2_. Phys. Rev. B.

[7] Kanna M., Wongnawa S., Buddee S., Dilokkhunakul K., Pinpithak P. (2010). Amorphous titanium dioxide: A recyclable dye remover for water treatment. J. Sol-Gel Sci. Technol..

[8] Hu S., Shaner M.R., Beardslee J.A., Lichterman M., Brunschwig B.S., Lewis N.S. (2014). Amorphous TiO_2_ coatings stabilize for Si, GaAs, and GaP photoanodes for efficient water oxidation. Science.

[9] Han C.H., Lee H.S., Lee K.W., Han S.D., Singh I. (2009). Synthesis of Amorphous Er^3+^-Yb^3+^ Co-doped TiO_2_ and Its Application as a Scattering Layer for Dye-sensitized Solar Cells. Bull. Korean Chem. Soc..

[10] Buddee S., Wongnawa S., Sirimahachai U., Puetpaibool W. (2011). Recyclable UV and visible light photocatalytically active amorphous TiO_2_ doped with M (III) ions (M = Cr and Fe). Mater. Chem. Phys..

[11] Pham H.H., Wang L.W. (2015). Electronic structures and current conductivities of B, C, N and F defects in amorphous titanium dioxide. Phys. Chem. Chem. Phys..

[12] Ghuman K.K., Singh C.V. (2013). Effect of doping on electronic structure and photocatalytic behavior of amorphous TiO_2_. J. Phys. Condens. Matter.

[13] Schneider J., Matsuoka M., Takeuchi M., Zhang J., Horiuchi Y., Anpo M., Bahnemann D.W. (2014). Understanding TiO_2_ Photocatalysis: Mechanisms and Materials. Chem. Rev..

[14] Huang Y., Ho W., Lee S., Zhang L., Li G., Yu J.C. (2008). Effect of Carbon Doping on the Mesoporous Structure of Nanocrystalline Titanium Dioxide and Its Solar-Light-Driven Photocatalytic Degradation of NOx. Langmuir.

[15] Qi D., Xing M., Zhang J. (2014). Hydrophobic Carbon-Doped TiO_2_/MCF-F Composite as a High Performance Photocatalyst. J. Phys. Chem. C.

[16] Inagaki M., Kojin F., Tryba B., Toyoda M. (2005). Carbon-coated anatase: The role of the carbon layer for photocatalytic performance. Carbon.

[17] Lee S., Lee Y., Kim D.H., Moon J.H. (2013). Carbon-Deposited TiO_2_ 3D Inverse Opal Photocatalysts: Visible-Light Photocatalytic Activity and Enhanced Activity in a Viscous Solution. ACS Appl. Mater. Interfaces.

[18] He D., Li Y., Wang I., Wu J., Yang Y., An Q. (2017). Carbon wrapped and doped TiO_2_ mesoporous nanostructure with efficient visible-light photocatalysist for NO removal. Appl. Surf. Sci..

[19] Shao Y., Cao C., Chen S., He M., Fang J., Chen J., Li X.F., Li D.Z. (2015). Investigation of nitrogen doped and carbon species decorated TiO_2_ with enhanced visible light photocatalytic activity by using chitosan. Appl. Catal. B..

[20] Park Y., Kim W., Park H., Tachikawa T., Majima T., Choi W. (2009). Carbon-doped TiO_2_ photocatalyst synthesized without using an external carbon precursor and the visible light activity. Appl. Catal. B..

[21] Nikkanen J.-P., Kanerva T., Mäntylä T. (2007). The effect of acidity in low-temperature synthesis of titanium dioxide. J. Cryst. Growth.

[22] Shao P., Tian J., Zhao Z., Shi W., Gao S., Cui F. (2015). Amorphous TiO_2_ doped with carbon for visible light photodegradation of rhodamine B and 4-chlorophenol. Appl. Surf. Sci..

[23] Kojima T., Sugimoto T. (2008). Formation Mechanism of Amorphous TiO_2_ Spheres in Organic Solvents 3. Effects of Water, Temperature, and Solvent Composition. J. Phys. Chem. C.

[24] Abdullah A.M., Al-Thani N.J., Tawbi K., Al-Kandari H. (2016). Carbon/nitrogen-doped TiO_2_: New synthesis route, characterization and application for phenol degradation. Arabian J. Chem..

[25] Morales M.A., Fernandez-Cervantes I., Agustin-Serrano R., Anzo A., Sampedro M.P. (2016). Patterns formation in ferrofluids and solid dissolutions using stochastic models with dissipative dynamics. Eur. Phys. J. B.

[26] Maletic M., Vukcevic M., Kalijadis A., Jankovic-Castvan I., Dapcevic A., Lausevic Z., Laušević M. (2016). Hydrothermal synthesis of TiO_2_/carbon composites and their application for removal of organic pollutants. Arabian J. Chem..

[27] Sing K.S.W., Everett D.H., Haul R.A.W., Moscou L., Pierotti R.A., Rouquerol J., Siemieniewska T. (1985). Reporting physisorption data for gas/solid systems with special reference to the determination of surface area and porosity (Recommendations 1984). Pure Appl. Chem..

[28] Factorovich M., Guz L., Candal R. (2011). N-TiO_2_: Chemical Synthesis and Photocatalysis. Adv. Phys. Chem..

[29] Etacheri V., Michlits G., Seery M.K., Hinder S.J., Pillai S.C. (2013). A Highly Efficient TiO_2_−xCx Nano-heterojunction Photocatalyst for Visible Light Induced Antibacterial Applications. ACS Appl. Mater. Interfaces.

[30] Palanivelu K., Im J.S., Lee Y.S. (2007). Carbon Doping of TiO_2_ for Visible Ligtht Photo Catalysis—A Review. Carbon Sci..

[31] Enache C.S., Schoonman J., van de Krol R. (2006). Addition of carbon to anatase TiO_2_ by n-hexane treatment—Surface or bulk doping?. Appl. Surf. Sci..

[32] Ebraheem S., El-Saied A. (2013). Band Gap Determination from Diffuse Reflectance Measurements of Irradiated Lead Borate Glass System Doped with TiO_2_ by Using Diffuse Reflectance Technique. Mater. Sci. Appl..

[33] Rochkind M., Pasternak S., Paz Y. (2015). Using Dyes for Evaluating Photocatalytic Properties: A Critical Review. Molecules.

[34] Wu T., Liu G., Zhao J., Hidaka H., Serpone N. (1998). Photoassisted Degradation of Dye Pollutants. V. Self-Photosensitized Oxidative Transformation of *Rhodamine B* under Visible Light Irradiation in Aquos TiO_2_ Dispertions. J. Phys. Chem. B.

[35] Pan L., Zou J.-J., Liu X.-Y., Liu X.-J., Wang S., Zhang X., Wang L. (2012). Visible-Light-Induced Photodegradation of Rhodamine B over Hierarchical TiO_2_: Effects of Storage Period and Water-Mediated Adsorption Switch. Ind. Eng. Chem. Res..

[36] Luna-Flores A., Valenzuela M.A., Luna-López J.A., Hernández de la Luz A.D., Muñoz-Arenas L.C., Méndez-Hernández M., Sosa-Sánchez J.L. (2017). Synergetic Enhancement of the Photocatalytic Activity of TiO_2_ with Visible Light by Sensitization Using a Novel Push-Pull Zinc Phthalocyanine. Int. J. Photoenergy.

