# Supplementary material for: An Easy-Made, Economical and Efficient Carbon-Doped Amorphous TiO2 Photocatalyst Obtained by Microwave Assisted Synthesis for the Degradation of Rhodamine B
Source: Materials (Basel). 2017 Dec 20;10(12):1447. doi: 10.3390/ma10121447 (PMC5744382; doi:10.3390/ma10121447)
Supplement: Supplementary file 1 [file materials-10-01447-s001.pdf]

materials
MDPI

Supplementary

An Easy-made, Economical and Efficient Carbon-doped Amorphous TiO_2 Photocatalyst Obtained by Microwave Assisted Synthesis for the Degradation of Rhodamine B

Adan Luna-Flores [1], José L. Sosa-Sánchez [2,*], Marco Antonio Morales-Sánchez [1], Ricardo Agustín-Serrano [3] and J. A. Luna-López [2]

[1] Facultad de Ingeniería Química-BUAP, Avenida San Claudio, Ciudad Universitaria, Puebla 72570, México; adan.luna@correo.buap.mx (A.L.-F.); spinor70@yahoo.com.mx (M.A.M.)
[2] Centro de Investigación en Dispositivos Semiconductores, Instituto de Ciencias BUAP, 14 sur y Avenida San Claudio, Ciudad Universitaria, A.P. 196, Puebla 72000, México; jose.luna@correo.buap.mx
[3] Centro Universitario de Vinculación y Transferencia de Tecnología OTC-BUAP, Prolongación de la 24 Sur y Avenida San Claudio, Ciudad Universitaria, Puebla 72570, México; ricardoagustin_s@hotmail.com
* Correspondence: jose.sosa@correo.buap.mx; Tel.: +52-222-2295500 (ext. 7872)

Received: 12 October 2017; Accepted: 11 December 2017; Published: 20 December 2017

DTiB-01

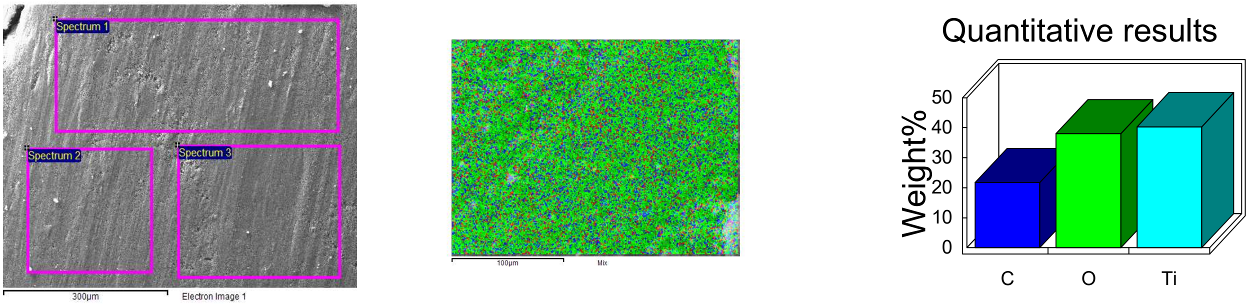

DTiB-02

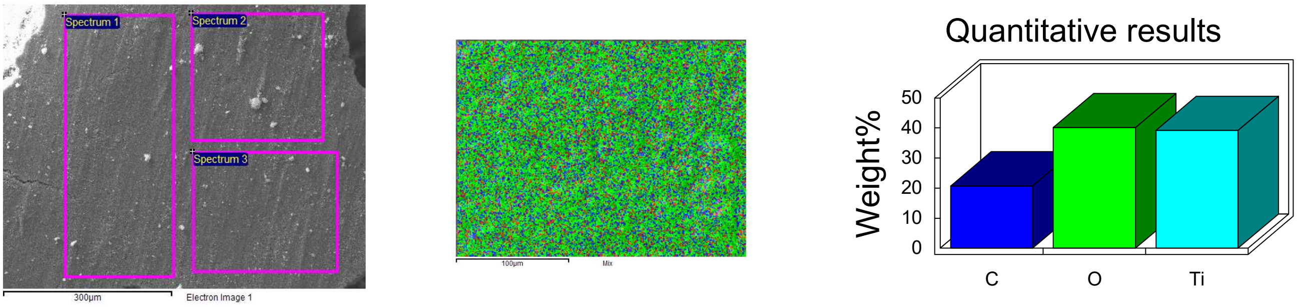

DTiB-03

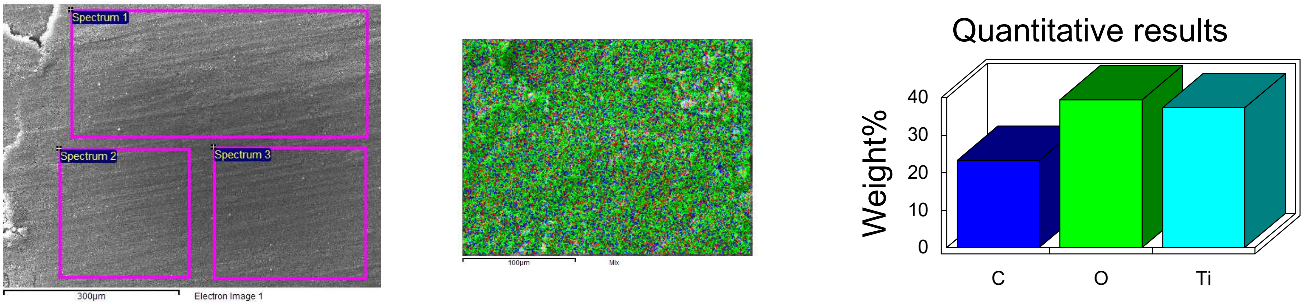

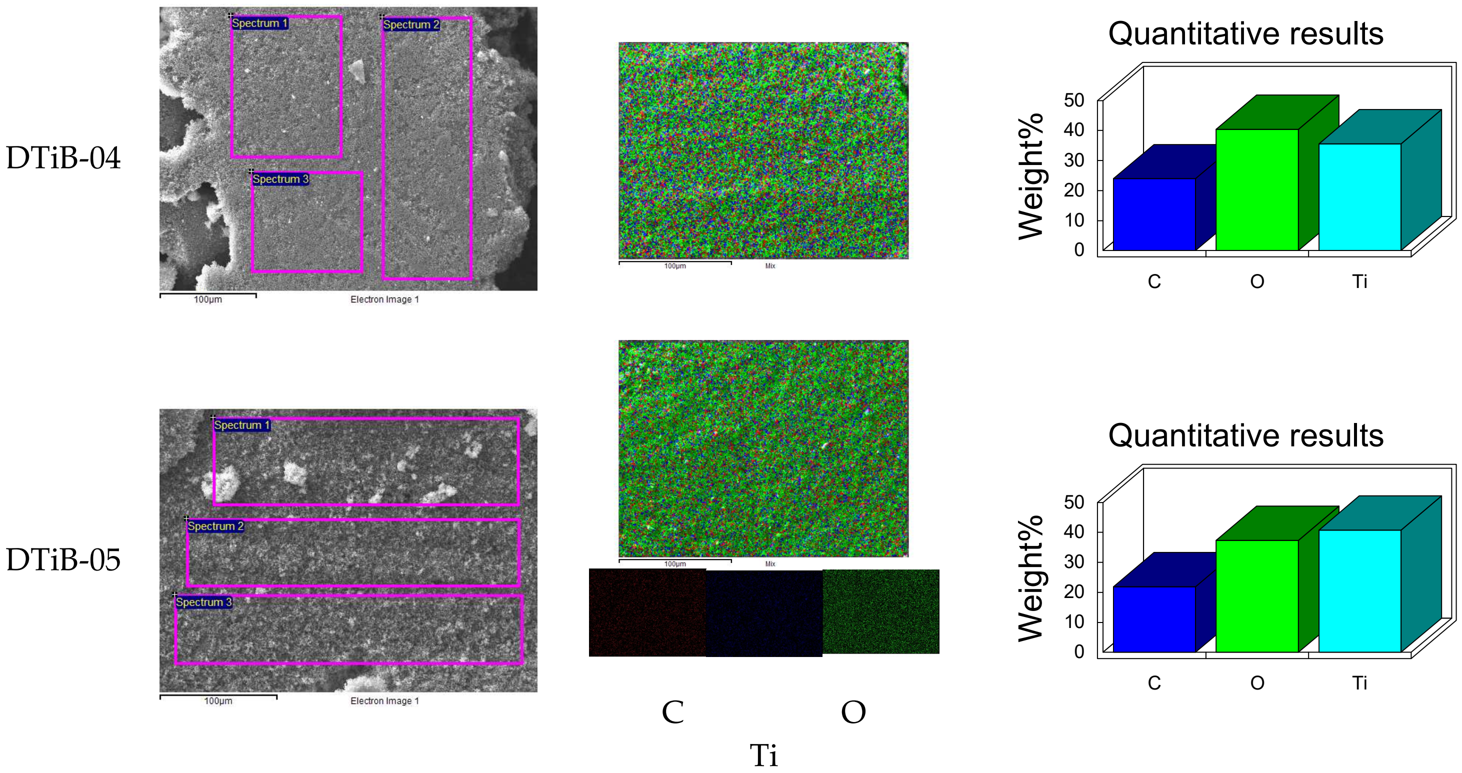

Figure S1. Elemental mapping from the EDS analysis of all the photocatalyst samples prepared in this work.

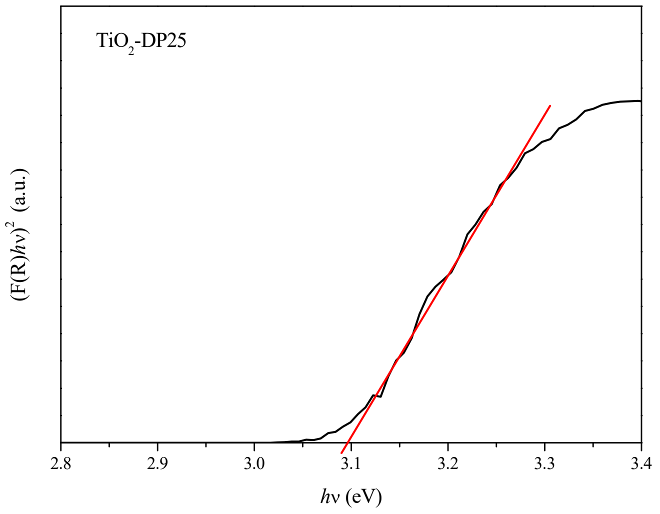

Figure S2. The relation between energy (hν, eV) and $(F(R)h\nu)^2$ for TiO_2-DP25.

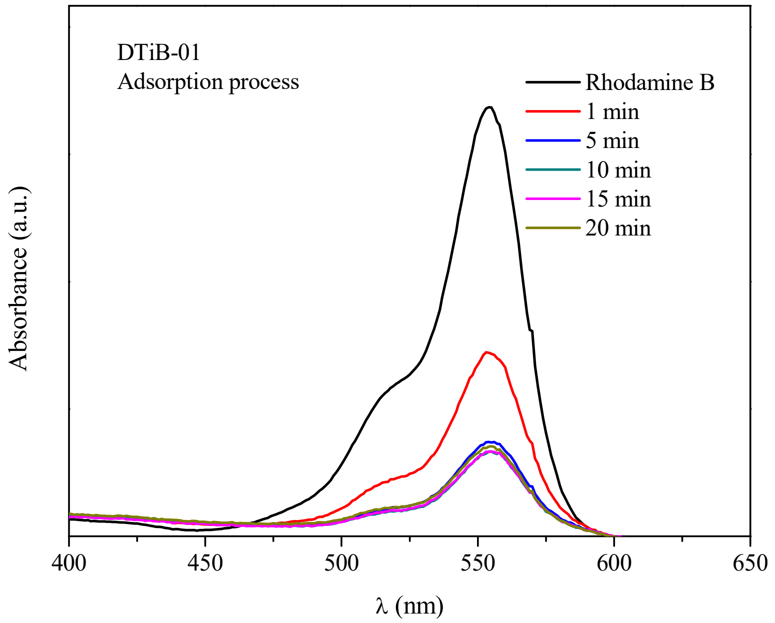

(a)

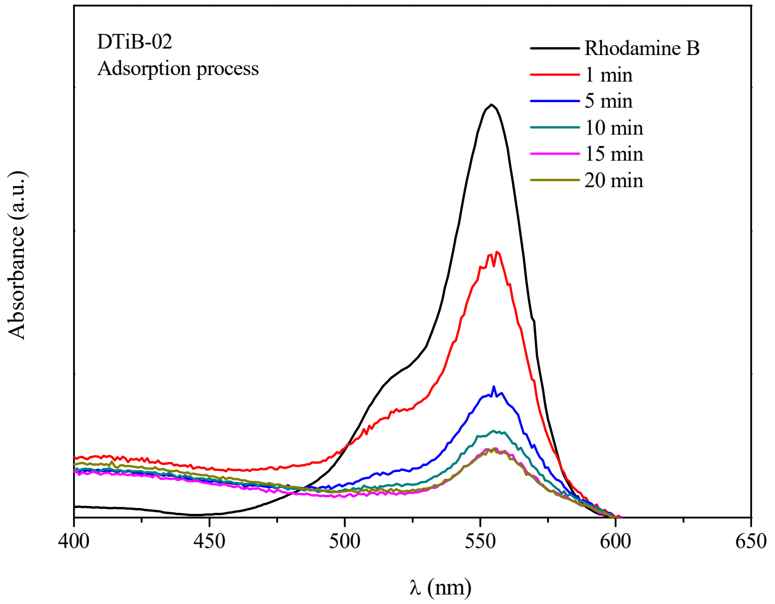

(b)

Figure S3. UV-vis spectra for the monitoring of the absorption of Rhodamine B in the dark onto the catalyst surface for the CD-aTiO_2 samples prepared.

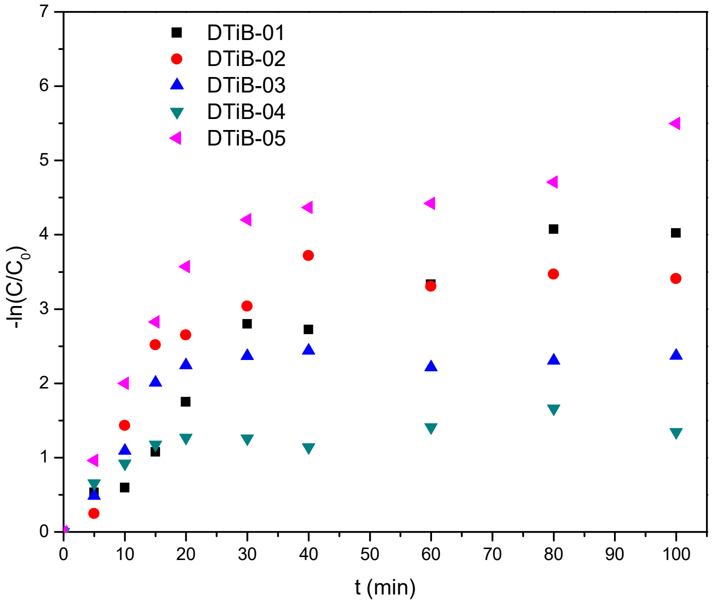

Figure S4. $-\ln(C/C_0)$ vs t plot in which it is apparent that the Rhodamine B degradation data do not fit for a pseudo first order kinetic equation.

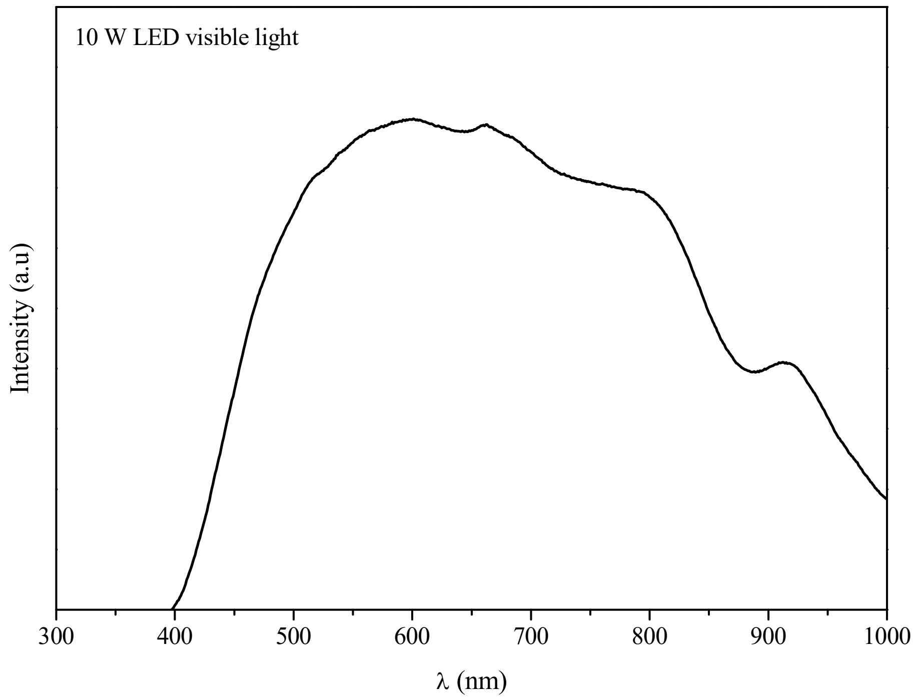

Figure S5. Emission spectrum for the lamp used in the photocatalytic experiments with visible light.

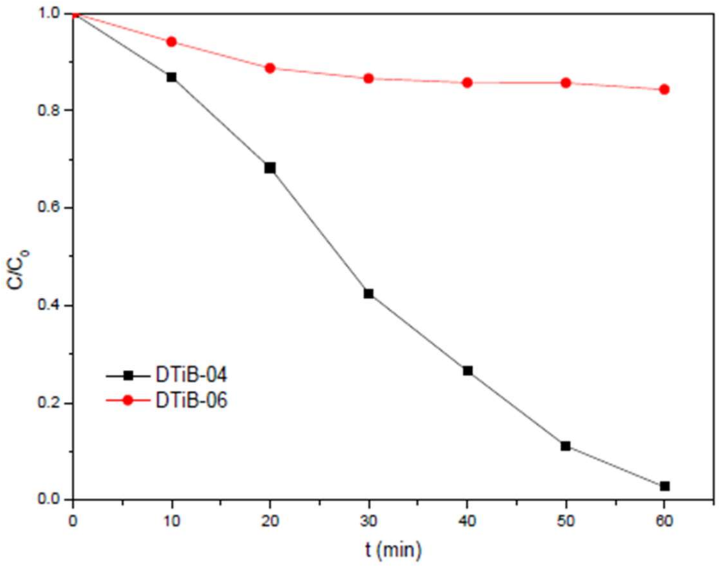

Figure S6. Comparison of the Rhodamine B solution (20 mg/L) degradation using a carbon doped TiO2 photocatalyst (DTiB-04) and another TiO2 material without carbon (DTiB-06).

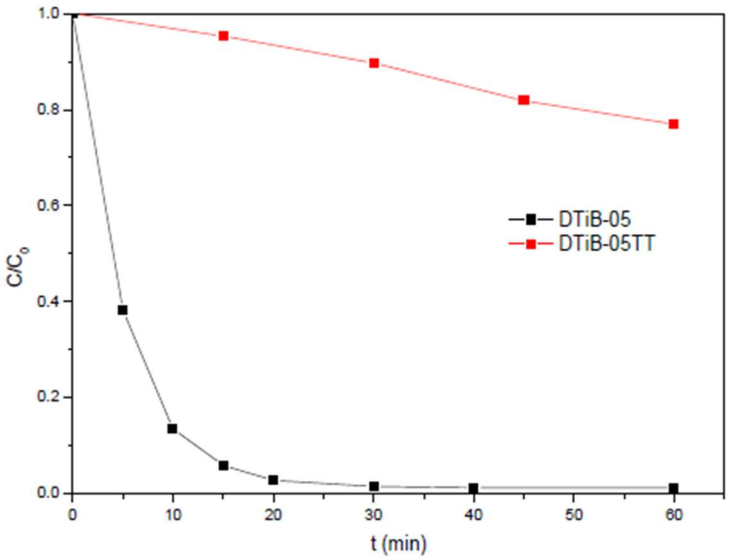

Figure S7. Rhodamine B solution (5 mg/L) degradation plots for the DTiB-05 and the DTiB-05TT photocatalysts.

© 2017 by the authors. Submitted for possible open access publication under the terms and conditions of the Creative Commons Attribution (CC BY) license (http://creativecommons.org/licenses/by/4.0/).
